# Post-traumatic ventricular septal defect manifesting as intermittent third-degree atrioventricular block: a case report

**DOI:** 10.1093/ehjcr/ytaf172

**Published:** 2025-04-08

**Authors:** Martin Benedikt, Martin Manninger, Anna-Sophie Eberl, Dirk von Lewinski, Daniel Scherr

**Affiliations:** Division of Cardiology, Department of Internal Medicine, Medical University of Graz, Auenbruggerplatz 15, 8036 Graz, Austria; Division of Cardiology, Department of Internal Medicine, Medical University of Graz, Auenbruggerplatz 15, 8036 Graz, Austria; Division of Cardiology, Department of Internal Medicine, Medical University of Graz, Auenbruggerplatz 15, 8036 Graz, Austria; Division of Cardiology, Department of Internal Medicine, Medical University of Graz, Auenbruggerplatz 15, 8036 Graz, Austria; Division of Cardiology, Department of Internal Medicine, Medical University of Graz, Auenbruggerplatz 15, 8036 Graz, Austria

**Keywords:** Atrioventricular block, Ventricular septal defect, Tricuspid regurgitation, CIED, Case report

## Abstract

**Background:**

Traumatic cardiac injuries are rare, but patients may present with symptoms like arrhythmias, heart failure, or cardiogenic shock.

**Case summary:**

A 50-year-old Caucasian construction worker was admitted to our emergency department with a new-onset third-degree atrioventricular (AV) block following a traumatic blunt chest injury at work. The arrhythmia was controlled by a continuous application of isoprenaline. After stabilization, the electrocardiogram showed sinus rhythm with a new-onset left bundle branch block. Transthoracic echocardiography revealed a ventricular septal defect, which could be confirmed by transoesophageal echocardiography, including a contrast study; however, the patient was initially rejected for acute cardiac surgery due to haemodynamic stable conditions. After several hours, the patient developed acute dyspnoea with pulmonary oedema and cardiogenic shock. Echocardiography revealed severe tricuspid regurgitation caused by rupture of the anterior papillary muscle, and the patient was immediately transferred to the department for cardiac surgery for acute ventricular septal patch plastic and tricuspid valve replacement. Post-surgery, the patient developed haemodynamically compromising third-degree AV block, required catecholamines and temporary transvenous pacing. A permanent pacemaker implantation was performed on the following day.

**Discussion:**

Mechanical complications after blunt chest injury are rare and surgical repair in unstable conditions are still the treatment of choice. In concomitant conduction disorders, close monitoring for arrythmias is obligatory in the early phase; however, implantation of a permanent pacemaker is often necessary.

**Learning points**

- Cardiac contusion is a rare cause of cardiac conduction disturbances and commonly presents with arrhythmias, heart failure, or cardiogenic shock.
- Transient or permanent cardiac conduction disturbances can be present after blunt chest injury and close monitoring should be initiated within the early phase.
- Routine use of contrast agents can help to confirm the diagnosis if echocardiography is suspicious or the diagnosis remains unclear.

## Introduction

Ventricular septal defect (VSD) is a rare complication following blunt chest trauma. It commonly occurs in the apical part of the muscular interventricular septum by anteroposterior compression during early systole, when the ventricles are maximally filled.^1^ Rupture of the interventricular septum occurs immediately after injury or delayed between 2 and 6 days, whereat delayed defects are more frequently associated with secondary myocardial necrosis following traumatic coronary damage.^2^ All heart chambers can be affected by blunt traumatic injury resulting in cardiac perforation; however, the right ventricle is the most common area of injury^3^ often associated with disruption of the tricuspid valve.^1^ Traumatic damage of myocardial fibres and intercellular electrical connections followed by myocardial oedema is well known to be the cause of the following structural and electrical complications. Atrioventricular block or bundle branch blocks tend to be transient after traumatic cardiac injury and commonly resolve by decrease in oedema; however, if conduction disturbances stay permanently, implantation of a pacemaker is often necessary.^4,5^

Treatment of the VSD is traditionally restricted to surgical repair, depending on the size of the VSD as well as haemodynamic condition of the patients.^6^

We report a case of a patient presenting with VSD and concomitant third-degree atrioventricular block after blunt chest injury.

## Summary figure

**Table:** 

Day 0–16:00	Traumatic cardiac injury and preclinical stabilization of haemodynamically compromising third-degree AV-Block
Day 0–17:00	Admission to ER with stable SR, Tapering of Isoprenaline, after taper new-onset LBBB
Day 0–17:30	TTE + TOE including contrast study, diagnosis of VSD, consultation of cardiac surgeon → conservative treatment
Day 1–02:00	Acute respiratory distress with lung oedema and cardiogenic shock
Day 1–02:30	Stabilization and diagnosis of ruptured anterior papillary muscle of tricuspid valve, consultation of cardiac surgeon
Day 1–03:00	Admission to the OR for valve replacement and patch plastic
Day 1–08:00	Transfer to ICU
Day 1–18:00	Echo control → orthotopic ventricular patch without shunt and normal prosthetic function
Day 6 Day 7–08:00	Extubation Start weaning of catecholamines
Day 7–20:00	Recurrence of haemodynamically compromising third-degree AV-Block, application of catecholamines followed by transvenous passagere pacing
Day 8	Implantation of dual-chamber pacemaker
Day 9	Transfer to cardiological ward, pacemaker control showing normal function, X-ray with normal lead-position
Day 12	Discharge from hospital
1 year post-traumatic injury	Asymptomatic, normal pacemaker function, high RV-pacing rate, normal cardiac function with normal prosthetic function, and orthotopic ventricular patch

ER, emergency room; SR, Sinus rhythm; LBBB, left bundle branch block; TTE, transthoracic echocardiography; TOE, transesophageal echocardiography; VSD, ventricular septal defect; OR, operating room; ICU, intensive care unit; AV, atrioventricular; RV, right ventricular

## Case presentation

**Figure 1:**
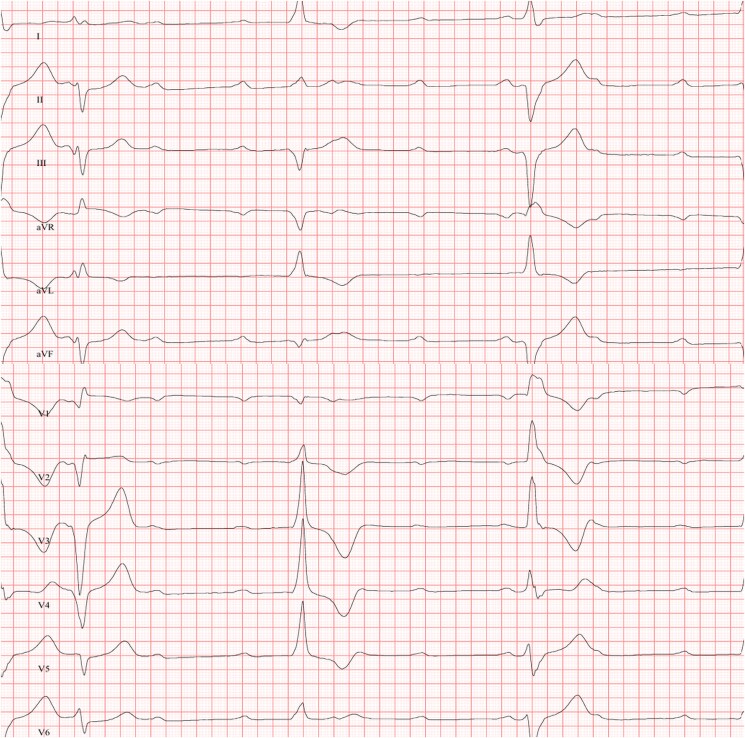
Initial electrocardiogram. Third-degree atrioventricular block with AV-dissociation and ventricular escape rhythm (©Medical University of Graz).

A 50-year-old Caucasian male construction worker was admitted to our emergency department after suffering a blunt chest injury, caused by a 200 kg concrete wall accidently falling on his chest at work, and compressing his thorax for several seconds. The patient presented with retrosternal pain and signs of dyspnoea. Physical examination of the thorax revealed numerous contusions as well as a pronounced ecchymosis on the upper chest, and the presence of bradycardia. Auscultatory there was no obvious pathological heart murmur, and the patient showed no signs of acute heart failure including increased jugular venous pressure or oedema. A 12-leads electrocardiogram showed a new-onset third-degree atrioventricular block (AVB III) with alternating ventricular escape rhythm of 45 beats per minute and AV-Dissociation (*Figure 1*). Nevertheless, the patient initially remained haemodynamically stable. The patient did not have a known past medical history, and he was not on any long-term medications. In order to increase heart rate, isoprenaline was administered with a dose of 1 µg/min continuously.

**Figure 2:**
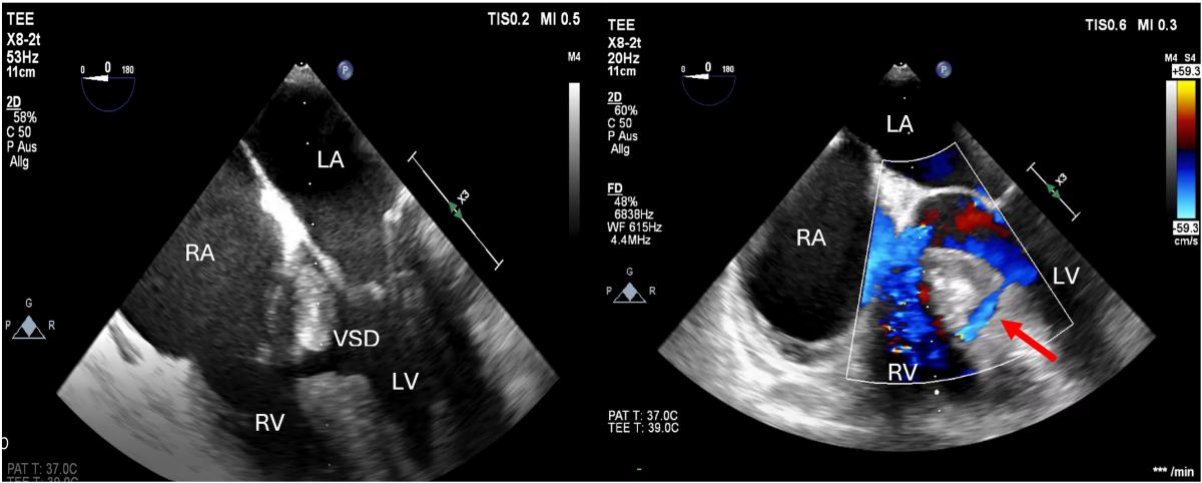
Transoesophageal echocardiography. Interventricular septal defect with left–right shunt (marked by red arrow); RA, right atrium; LA, left atrium; LV, left ventricle; RV, right ventricle; VSD, ventricular septal defect (©Medical University of Graz).

After arrival at the intensive care unit (ICU), the patient presented in stable sinus rhythm with 1:1 AV-conduction and new-onset left bundle branch block, hence, isoprenaline was tapered successfully. Biomarker levels, including a creatinine kinase of 1,832 U/L, a troponin T of 1,910 pg/mL, as well as a myoglobin of 200 ng/mL were elevated due to damaged skeletal muscle and injured cardiac tissue. Transthoracic echocardiography (TTE) revealed normal systolic function without valvular disease or pericardial effusion/tamponade, but a ventricular mid-septal defect of 8–9 mm diameter with left–right shunting was observed; however, no signs of right ventricular volume overload were detected. Transoesophageal echocardiography (TOE), including a contrast study, was performed, confirming the diagnosis of the VSD by an early systolic pass of contrast agent into the right ventricle (*Figure 2*). Pneumothorax and pulmonary embolism were ruled out by a computer tomography scan, which showed fractures of the third to sixth ribs on both sides.

The case was initially presented to the clinical department for cardiac surgery and further discussed in the interdisciplinary heart team. The patient was rejected for immediate ventricular patch plastic due to haemodynamically stable conditions, as well as the recent onset of the traumatic VSD, and therefore, surgery was scheduled for the following days.

**Figure 3:**
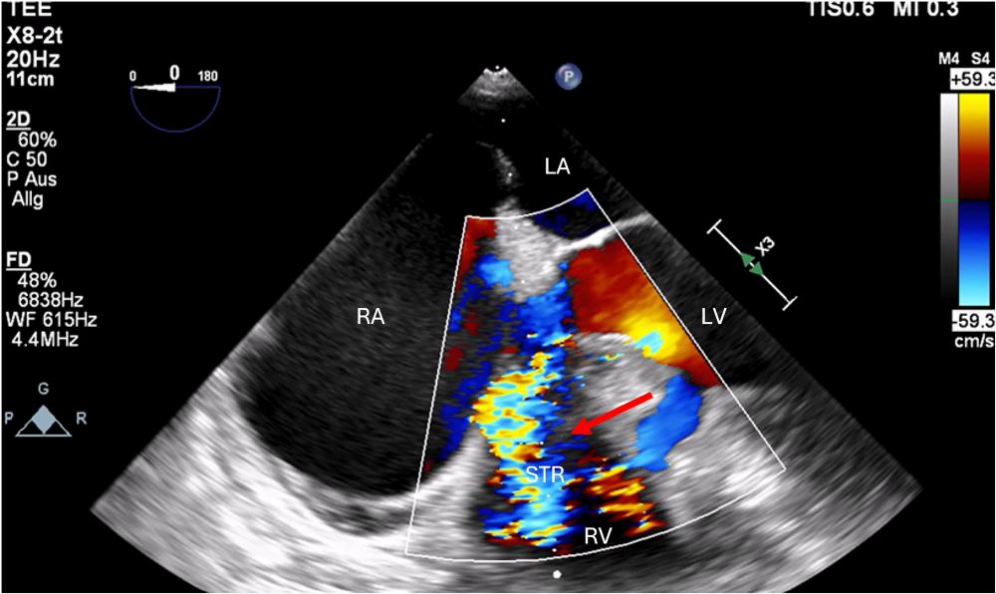
Transoesophageal echocardiography. Severe tricuspid regurgitation (marked by red arrow) with dilation of the right heart chambers; RA, right atrium; LA, left atrium; LV, left ventricle; RV, right ventricle; STI, severe tricuspid regurgitation (©Medical University of Graz).

**Figure 4:**
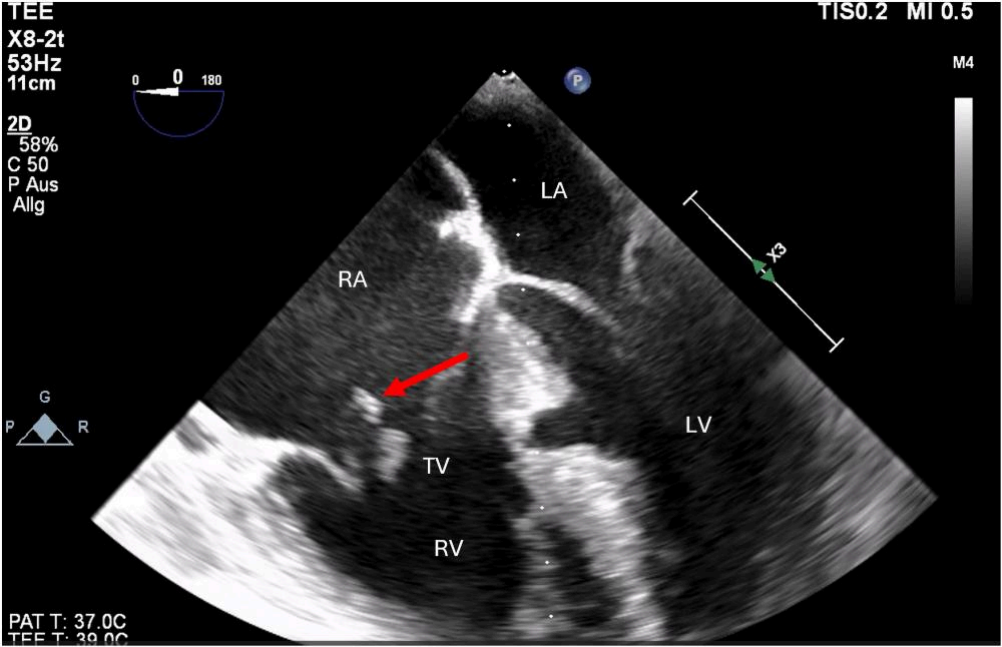
Transoesophageal echocardiography. Rupture of the tricuspid anterior papillary muscle (marked by red arrow); RA, right atrium; LA, left atrium; LV, left ventricle; RV, right ventricle; TV, tricuspid valve (©Medical University of Graz).

Within the following hours, the patient developed acute dyspnoea with progressive pulmonary oedema, and cardiogenic shock requiring stabilization with 0.2 mg/h noradrenalin and 80 mg furosemide. A new transthoracic echocardiography revealed severe dilatation of all heart chambers, and a new-onset severe tricuspid regurgitation (*Figure 3*; Supplementary material online, *Video S2*). Subsequent TOE verified a rupture of the anterior tricuspid papillary muscle (*Figure 4*; Supplementary material online, *Video S1*) with dilation of the inferior vena cava.

After stabilization, immediate transfer to the operating room was organized, and ventricular septal patch plastic, as well as tricuspid valve replacement by a biological prothesis, was performed. During follow-up at the ICU, the patient always remained haemodynamically stable under low-dose catecholamines and was weaned from the respirator after 5 days.

Transthoracic echocardiography displayed normal tricuspid prosthetic function without paravalvular regurgitation, and orthotopic located ventricular patch without signs of left–right shunting. Catecholamines were reduced stepwise until continuously application could be stopped; however, after several hours, the patient developed recurrent haemodynamically compromising third-degree AVB again, requiring catecholamines as well as temporary transvenous pacing, and dual-chamber pacemaker implantation was performed the following day.

One day post-surgery, the patient was transferred to the cardiological ward in stable conditions. Chest X-ray revealed normal lead positioning without signs for pneumothorax, and interrogation of the pacemaker showed normal device function.

The patient was discharged from hospital after 3 days. Annually follow-up visits in our out-patient clinic were scheduled showing normal pacemaker function as well as adequate function of the prosthetic valve and ventricular patch. Further telemedical monitoring was established, and the patient was registered for cardiological rehabilitation.

## Discussion

Traumatic thoracic injury can be associated with severe structural cardiac diseases, and is a rare cause of cardiac conduction diseases, in which bundle branch block and high-degree AVB are reported to be the most common conduction diseases following blunt cardiac injuries.^7,8^ Although traumatic cardiac injuries are rare, the clinical presentation can range from incomplete BBB or low-degree AV-Block to complete heart block. Most of the post-traumatic conduction disorders are transient and self-terminating after the decrease of myocardial oedema; however, a small percentage of patients suffer from persistent conduction diseases requiring implantation of a permanent pacemaker due to rupture of myocardial fibres and electrical connective tissue, followed by pronounced myocardial necrosis.^4,5^ Reversibility of conduction disturbances after traumatic VSDs might depend on the location of the myocardial injury. Whereas basal and mid-septal VSDs, located in the membranous and peri-membranous part of the septum, appear to have a higher risk of developing permanent conduction diseases due to the proximity to the conduction system and the His-Bundle, apical VSDs might have lower risk of permanent conduction disturbances; however, clinical data in this area are missing.^9,10^

Treatment of VSD depends on defect size and haemodynamic condition; however, in most cases, a surgical repair is commonly preferred.^6^ Whereas small VSDs, commonly located in the muscular or peri-membranous part of the septum, can be treated conventionally or with percutaneous closure systems, large VSDs, with a diameter of >10 mm diameter, require surgical treatment, especially when haemodynamically compromising or valves are involved.^11,12^ Depending on the haemodynamic behaviour of the VSD, delayed surgical repair is favoured aiming a primary stabilization of the patients within the first days. This goes in line with the injured myocardium being still friable or necrotic during patch repair, and multiple case reports highlight repair failure in the early phase, with the need for surgical revisions.^11^ Hence, delayed surgical repair allows the injured myocardium to perform fibrotic modification within the first few days to increase post-interventional prognosis.^11^

This case report emphasizes the awareness of traumatic cardiac complications after blunt cardiac injury, which can occur immediately, but also several hours or days after the event. Clinical manifestation includes severe arrhythmias, acute heart failure, or cardiogenic shock. This case also highlights the importance of echocardiography to identify severe cardiac complications, and the additional use of contrast agents when diagnosis remains unclear.

One question of interest would be the use of conduction system pacing (CSP) in these patients. In our case, the use of CSP was primarily discussed; however, the RV lead was implanted conventionally in the lower mid-septal area due to the large VSD and the risk for lead dysfunction in a pacemaker-dependent patient. Previous trials reported the safety and feasibility of CSP in patients with congenital heart disease (CHD), although implantation is complex.^13^ His-Bundle pacing was reported to be effective in adults with atrioventricular canal defect.^14^ A small single-centre series, including 24 patients, demonstrated a high success rate of His-Bundle Pacing in paediatric and CHD including ventricular septal repair, without peri-procedural complications.^15^ Additionally, a small single-centre study reported successful performance of left bundle branch area pacing in CHD patients including VSDs.^16^ Although CSP appears to be safe and feasible in VSD patients, the benefit over conventional pacing in VSD remains unclear.

## Conclusion

Mechanical complications after blunt chest injury are rare, and surgical repair is still the treatment of choice. In concomitant conduction disorders, permanent pacemaker implantation is often necessary; however, data on using CSP in these patients is still scarce and further research in this area needs to be performed.

## Supplementary Material

ytaf172_Supplementary_Data

## Data Availability

The data underlying this article are available in the article and its online Supplementary material.

## Lead author biography

Dr Martin Benedikt received his Medical Doctor (MD) in 2020 at the Medical University of Graz and is currently a trainee for cardiology at the Clinical Department for Cardiology at the Medical University of Graz. His research includes the use of anti-diabetics in cardiovascular diseases as well as cardiac electrophysiology and device therapy.

## Supplementary material

**Consent**: The patient expressed his consent to add to the current scientific knowledge to help the cardiology community and other affected patients. This case follows the Guidelines of the Committee on Publication Ethics (COPE).

**Funding**: D.V.L. is supported by the Austrian Science Fund (FWF) grant KLIF-1155-B.
